# Molecular Identification and Sequencing of Mannose Binding Protein (MBP) Gene of *Acanthamoeba palestinensis*

**Published:** 2010-03

**Authors:** M Niyyati, S Rezaie, Z Babaei, M Rezaeian

**Affiliations:** Department of Parasitology and Mycology, School of Public Health, Tehran University of Medical Sciences, Tehran, Iran

**Keywords:** *Acanthamoeba palestinensis*, Mannose Binding Protein (MBP), PCR

## Abstract

**Background:**

*Acanthamoeba* keratitis develops by pathogenic *Acanthamoeba* such as *A. palestinensis*. Indeed this species is one of the known causative agents of amoebic keratitis in Iran. Mannose Binding Protein (MBP) is the main pathogenicity factors for developing this sight threatening disease. We aimed to characterize MBP gene in pathogenic *Acanthamoeba* isolates such as *A. palestinensis*.

**Methods:**

This experimental research was performed in the School of Public Health, Tehran University of Medical Sciences, Tehran, Iran during 2007-2008. *A. palestinensis* was grown on 2% non-nutrient agar overlaid with *Escherichia coli*. DNA extraction was performed using phenol-chloroform method. PCR reaction and amplification were done using specific primer pairs of MBP. The amplified fragment were purified and sequenced. Finally, the obtained fragment was deposited in the gene data bank.

**Results:**

A 900 bp PCR-product was recovered after PCR reaction. Sequence analysis of the purified PCR product revealed a gene with 943 nucleotides. Homology analysis of the obtained sequence showed 81% similarity with the available MBP gene in the gene data bank. The fragment was deposited in the gene data bank under accession number **EU678895, **

**Conclusion:**

MBP is known as the most important factor in *Acanthamoeba* pathogenesis cascade. Therefore, characterization of this gene can aid in developing better therapeutic agents and even immunization of high-risk people.

## Introduction

A*canthamoeba* spp. is a ubiquitous free–living amoeba in environmental habitats including air, dust, water, sewage, and hospital wards (1, 2). Pathogenic genotypes mainly T4 genotype could affect the central nervous system or cornea which sometimes leads to death and blindness, respectively (1–4).

Our previous study revealed that in Iran T2, T3, T4 and T11 genotypes were the causative agents of *Acanthamoeba* keratitis (AK) (5). Molecular analysis of environmental samples including soil, water and cow feces in Iran have also showed T4 genotype as a predominate genotype in this region (5). It should be mentioned that accurate and early diagnosis of AK is very important for a good outcome of disease (6). To date, it has been proven that an important protein involved in pathogenesis of AK is the Mannose Binding Protein (MBP) (7). MBP is a transmembrane protein, which is located on the surface of the amoeba and acts as a receptor for mannose residues on corneal glycoproteins (8). Soft contact lenses as well as corneal trauma are the two major factors for developing AK (8). Corneal trauma could develop wearing contact lenses. Besides, corneal trauma is known as a predisposing factor in the up-regulation of mannosylated glycoproteins (7, 8). In our previous study, we isolated and characterized the gene encoding MBP of an Iranian isolate of *A. castellanii*. This study showed that 1081 bp of this gene encoded a protein with 194 amino acids (9).

Since, characterization and sequencing of this important gene in the pathogenic *Acanthamoeba* strains can open the way for further investigation such as developing better therapeutic agent as well as immunization, we decided to characterize this gene in pathogenic *Acanthamoeba* isolates such as *A. palestinensis*.

## Materials and Methods

### Parasite

*A. palestinensis* isolated from a keratitis patient in the Department of Parasitology and Mycology, School of Public Health, Tehran University of Medical Sciences, Iran was our sample. The species was examined previously in Birckbeck College, London University (6).

### Culture

Amebas were cultured on 2% non-nutrient agar (NNA) along with Escherichia coli as a food source for amoeba according to our previous study (9). After a few days, many trophozoites were obtained. Acanthamoeba were harvested and washed three times with phosphate buffer saline (PBS) solution in order to eliminate agar.

### DNA extraction

Total genomic DNA was extracted by modified phenol-chlorophorm method (10). Briefly, the cells were resuspended in DNA lysis buffer including EDTA and Tris-HCl (pH 8.0) and in the next step addition of 3% SDS and proteinase K (10 mg/ml) were performed. This process continued with incubation at 55°C for two hours using phenol-chlorophorm-isoamyl alcohol (25:24:1) and chlorophorm–isoamyl alcohol (24:1). Finally, DNA was recovered by cold absolute ethanol and sodium acetate (3 M).

### PCR reaction

PCR amplification of a gene encoding MBP was performed by a pair of primers for a part of the MBP gene. These primers were designed based on the available MBP gene sequence in the gene data bank (7). Nucleotide sequences of these primers were as follows: 5'GTC TTG ATG GTG GCC TTG TT 3'as forward and 5'CCC ACA CCT CCT TGT CCT TA 3’ as reverse. It was estimated that these primers correspond to a 900 bp of MBP gene. The 50 µl reaction mixture consisted of 20 ?M forward and reverse primers, 20 mM PCR buffer (with MgCl_2_), (Roche, Germany), 0.2 mM deoxynucleoside triphosphates (dNTPs), 1.2 Unit Taq DNA polymerase (Cinnagene, Tehran, Iran), 1 µl template DNA.

PCR reaction was performed in theromocycler (Primus) through 35 cycles at 94°C for 30" (Denaturation), 52°C for 1:30 min (Annealing) and 72°C for 2 min (Extension) followed by last extension time of 10 min (72°C).

### Purification and Sequencing of PCR- Product

The PCR product was then sliced from the agarose gel and then purified by Qiagen kit (USA) in order to eliminate excess nucleotides, dimers and non specific bands and then submitted for sequencing to MWG-Germany.

## Results

**Fig. 1:**
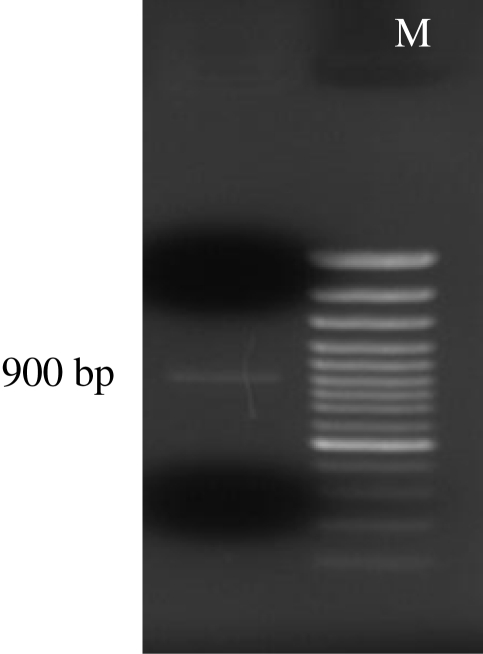
900 bp PCR- product of *Acanthamoeba palestinensis* MBP gene on 2% agarose gel M=Molecular Weight Marker (100 bp)

Total genomic DNA was detected on 2% agarose gel. A 900 bp PCR-product was also recovered after PCR reaction using UV transilluminator (Fig. 1). Sequence analysis of the purified PCR product revealed a gene with 943 nucleotides. Homology analysis of the obtained sequence using Basic Local Alignment Search Tool (BLAST) in the Gene Data Bank (NCBI, NIH) showed a 81% similarity with the available MBP genes in the Gene Data Bank (Accession numbers: **EU363513,AY604039**). This fragment encodes a protein with 148 amino acids. Homology analysis at the amino acid level revealed a low homology of the amplified fragment with other proteins in the Gene Data Bank. Besides, there were three introns within the amplified fragment. The data of this fragment was deposited in the Gene Data Bank, under the following accession number for public access: **EU678895**.Additionally, analysis of amino acid sequences revealed a high amount of serine, while, histidine was attributed to be the lowest amount of amino acids within this fragment.

## Discussion

We isolated and sequenced a part of gene encoding MBP in *A.* *castellanii* in our previous study (9). In the present study, we have sequenced and characterized a fragment of MBP gene in *A. palestinensis* as a causative agent of AK. Previous researches conducted in Iran regarding *Acanthamoeba* related keratitis showed that *A. palestinensis* (T2 genotype) could be an important causative agent of AK in Iran (6).

Pathogenesis cascade of *Acanthamoeba* involves a variety of complex processes. Many studies have shown that a carbohydrate recognition based process has a key role in this regard (1, 2). Indeed, the first crucial step in the pathogenicity process is the adherence of the trophozoite form of amoeba to epithelial corneal cells (1, 2). To date it has been shown that there are two main proteins involved in the adhesion of amoeba to corneal epithelium including MBP and Laminin Binding Protein (LBP) (2).

Studies regarding MBP have been started since 2004 and revealed that MBP is an important virulent pathogenicity factor (10). This protein also introduced as a protective antigen and studies on the animal models have shown that MBP could be a proper vaccine candidate for high-risk people especially within contact lens wearers (11). In fact, antibodies against MBP in tear film could inhibit the interaction between amoeba and corneal epithelium (12). Garate et al. reported that MBP gene contains 3620 bp and this gene encodes 833 amino acid (10). Despite, the report by Garate et al., which showed that MBP expression, was very low in *A. palestinensis*, this species has been reported as a causative agent of amoebic keratitis. Additionally, our result confirmed that *A. palestinensis* contains a gene encoding MBP which is the most important virulence factor.

Present study has revealed a 943 bp of the MBP gene in *A. palestinensis*. Homology analysis of this fragment has shown that there is 81% similarity between *A. palestinensis* and *A. castellanii* at the nucleotide level. The joint of exons was in 1 to15, 195 to251, 347 to 426, 652….>943.

In support of our study, previous homology analysis of MBP amino acids in *A. castellanii* showed a low homology with any other proteins (11, 12). We have shown that *A. palestinenis* also has a low similarity with other proteins using BLAST search tool. Indeed, it is worth to mention that the amplified fragment of MBP gene is a polymorphic region and therefore, other studies should be performed for identification of constant part of MBP gene as well as determination of functional domains.

In conclusion, MBP introduces as the most important factor in *Acanthamoeba* pathogenesis cascade and characterization of this gene in pathogenic *Acanthamoeba* can help researches for developing better therapeutic agents and even immunization of high-risk peoples in the near future.
